# Antiviral response and immunopathogenesis of interleukin 27 in COVID-19

**DOI:** 10.1007/s00705-023-05792-9

**Published:** 2023-06-13

**Authors:** Juan Felipe Valdés-López, Silvio Urcuqui-Inchima

**Affiliations:** Grupo Inmunovirología, Facultad de Medicina, Universidad de Antioquia UdeA, Calle 70 No. 52-21, Medellín, Colombia

**Keywords:** SARS-CoV-2, COVID-19, Innate immune response, Toll-like receptors, Inflammatory response, Antiviral response, Interleukin 27, Interferon

## Abstract

The coronavirus disease 2019 (COVID-19) pandemic caused by severe acute respiratory syndrome coronavirus 2 (SARS-CoV-2) infection is associated with a high mortality rate. The clinical course is attributed to the severity of pneumonia and systemic complications. In COVID-19 patients and murine models of SARS-CoV-2 infection, the disease may be accompanied by excessive production of cytokines, leading to an accumulation of immune cells in affected organs such as lungs. Previous reports have shown that SARS-CoV-2 infection antagonizes interferon (IFN)-dependent antiviral response, thereby preventing the expression of IFN-stimulated genes (ISGs). Lower IFN levels have been linked to more-severe COVID-19. Interleukin 27 (IL27) is a heterodimeric cytokine composed of IL27p28 and EBI3 subunits, which induce both pro- and anti-inflammatory responses. Recently, we and others have reported that IL27 also induces a strong antiviral response in an IFN-independent manner. Here, we investigated transcription levels of both IL27 subunits in COVID-19 patients. The results show that SARS-CoV-2 infection modulates TLR1/2-MyD88 signaling in PBMCs and monocytes and induces NF-κB activation and expression of NF-κB-target genes that are dependent on a robust pro-inflammatory response, including EBI3; and activates IRF1 signaling which induces IL27p28 mRNA expression. The results suggest that IL27 induces a robust STAT1-dependent pro-inflammatory and antiviral response in an IFN-independent manner in COVID-derived PBMCs and monocytes as a function of a severe clinical course of COVID-19. Similar results were observed in macrophages stimulated with the SARS-CoV-2 spike protein. Thus, IL27 can trigger an antiviral response in the host, suggesting the possibility of novel therapeutics against SARS-CoV-2 infection in humans.

## Introduction

Coronavirus disease 2019 (COVID-19), caused by severe acute respiratory syndrome coronavirus 2 (SARS-CoV-2), a single-stranded positive-sense RNA (ssRNA+) virus, is associated with a high mortality rate, which is attributed to the severity of pneumonia and systemic complications [reviewed in ref. 1]. Most SARS-CoV-2-infected individuals have mild flu-like symptoms, but about 20% of infected individuals develop a more severe illness due to viral pneumonia, often resulting in a need for hospitalization [2]. The clinical manifestations of COVID-19 can vary from asymptomatic infection to mild-moderate, severe, and ultimately, critical respiratory illness with multi-organ dysfunction that can lead to death [3]. Studies of COVID-19 patients and murine models of SARS-CoV-2 infection have indicated that severe disease may be triggered by excessive production of cytokines (cytokine storm), leading to an accumulation of immune cells in affected organs such as lungs [4, 5]. Hyperinflammation is associated with exacerbation of symptoms in severe and critical COVID-19 patients [6]. Huang et al. reported that SARS-CoV-2 infection generates a delayed but overactive production of interleukin (IL) 1β (IL1β), IL6, CXCL8/IL8, IL10, tumor necrosis factor alpha (TNFα), and monocyte chemoattractant protein 1 (CCL2/MCP1), inducing a dysregulated innate immune response [3]. Numerous studies have shown that markers such as IL6 and CXCL8/IL8, as well as certain patterns in multiomics-based studies, might be associated with COVID-19 severity and lethality [7–9]. However, although severe disease is often associated with elevated pro-inflammatory cytokines [10], given the clinical diversity and variable characteristics of the disease, it is unclear how dysregulation of innate and adaptive immune responses is linked to the severity of COVID-19.

The innate immune response is the first line of host defense against viral infection and is initiated by activation of pattern recognition receptors (PRRs), including Toll-like receptors (TLRs) [11], which are the central mediators of the innate and adaptive immune responses. TLRs recognize conserved pathogen-associated molecular patterns (PAMPs) of microbes to induce pro-inflammatory and antiviral responses [11–14]. The cell-surface TLR TLR2 forms a homodimer or a heterodimer with TLR1 or TLR6 (TLR2 complex) [15]. The TLR2 complex plays an essential role in sensing diverse microbial PAMPs, including lipoproteins, lipoteichoic acid (Gram-positive bacteria), and chitin (fungi) [16]. Upon recognition of PAMPs, TLR2 recruits adaptor proteins, including myeloid differentiation primary response 88 (MyD88) and interleukin 1 receptor-associated kinase (IRAK) to form a myddosome and initiate signaling pathways that culminate in activation of nuclear factor κB (NF-κB) and transcription factors, including NF-κB1, NF-κB2, RELA, RELB, c-REL, and IκBα (negative regulator) [11, 17, 18]. Activation of NF-κB results in the transcription of many NF-κB target genes that promote secretion of cytokines and chemokines that are involved in the pro-inflammatory response [17, 19–24]. Additionally, the TLR2 complex recognizes structural PAMPs of several viruses, including human immunodeficiency virus type 1 (HIV-1) [25], herpes simplex virus 1 [26], hepatitis C virus (HCV) [27], dengue virus [28], and chikungunya virus (CHIKV) [17]. Furthermore, it has been reported that TLR2 recognizes the SARS-CoV-2 envelope (E) and spike (S) glycoproteins to induce a pro-inflammatory response [29–31]. While there has been one study showing that TLR4 recognizes the SARS-CoV-2 spike protein to induce a strong immune response [32], another study showed that SARS-CoV-2 by itself was not recognized by TLR4 in TLR4-HEK293 cells or primary human dendritic cells [33].

RNA sensors such as TLR3 and TLR7 also play a crucial role in recognition of SARS-CoV-2 RNA, and the activation of signaling pathways that culminate in induction of NF-κB and interferon response factors (IRFs), including IRF1, IRF3, and IRF7 [17, 31, 34]. NF-κB and IRFs regulate expression of pro-inflammatory mediators as well as interferons (IFNs) in many cell types [11, 35, 36].

The antiviral response is closely associated with the transcription of interferon (IFN) genes, and their inhibition can lead to increased susceptibility to viral infections [37]. Based on their genetic, structural, and functional characteristics, as well as their receptor usage for signaling in cells, three types of IFNs have been identified [38–40]. Type I IFN (IFNα, β, ε, κ, and ω) binds to the IFN alpha/beta receptor complex (IFNAR, IFNAR1/IFNAR2) [39, 41], type II IFN (IFNγ) signals through the IFN-gamma receptor complex (IFNGR, IFNGR1/IFNGR2) [38], and type III IFN (IFNλ1–4) binds to the interferon-lambda receptor complex (IFNLR, IFNLR1/IL10RB) [42]. IFN-I/IFNAR and IFN-III/IFNLR are the most important IFNs/IFNRs expressed by many cell types and are secreted from infected cells. Thus, these two types of IFN trigger specific gene expression via the Janus kinase (JAK) signaling pathway, which phosphorylates and activates signal transducer and activator of the transcription 1 (STAT1) and STAT2, which, together with IRF9 form a transcription factor complex known as IFN-stimulated gene factor 3 (ISGF3) [37, 43]. ISGF3 is translocated into the nucleus and binds to IFN-stimulated response elements (ISREs) to upregulate the expression of numerous IFN-stimulated genes (ISGs) encoding antiviral proteins (AVPs), cytokines, and chemokines that maintain the antiviral state.

In infected cells, SARS-CoV-2 non-structural proteins inhibit innate immune receptor signaling and IFN production [44]. Furthermore, SARS-CoV-2 is highly sensitive to exogenously administered type I/III IFNs [45]. Hadjadj et al. reported low levels of IFN-I in COVID-19 patients, which correlated with poor outcomes and prognosis [46]. Moreover, suppression of the IFN-I and IFN-III response in patients is associated with severe COVID-19 [46–48]. It has been suggested that the weak IFN-dependent antiviral response in COVID-19 patients may be due to the failure of innate immune cells to mount an appropriate IFN-I response to infection, as evidenced by a reduced number and activity of plasmacytoid dendritic cells, the main source of IFNα during viral infections, in COVID-19 patients [49]. Additionally, it has been reported that the IFN-I-dependent antiviral response in lungs is stronger in patients with moderate disease than in those with severe COVID-19 [49]. IFN-III also plays an important role in the host defense against SARS-CoV-2 infection, since the production of IFNλ1 and IFNλ3 in the upper airway induces the expression of protective ISGs in mild COVID-19 cases [50]. However, it has been reported that SARS-CoV-2 infection appears to result in disruption of the IFN response [51], which is linked to a loss of function of genes that encode regulators of IFN induction, including TLR3, IRF3, IRF9, and IFN-I signaling [52]. Loske et al. reported that basal levels of several PRRs and ISG were pre-activated in children, but, upon SARS-CoV-2 infection, their expression was higher in children than in adults [53]. However, expression of the IFN coding genes was not investigated in those studies. Together, the data suggest that SARS-CoV-2 has evolved mechanisms to inhibit IFN and downstream signaling targets to escape host immunity. Thus, identification of factors that enhance the innate antiviral response that are independent of IFN signaling might contribute to the development of novel therapeutics against COVID-19.

Interleukin 27 (IL27) is a heterodimeric cytokine belonging to the IL6/IL12 family of cytokines that is composed of IL27p28 and Epstein-Barr virus-induced 3 (EBI3) subunits [54]. Its interaction with a heterodimer compound of IL27Rα and Gp130 [55] activates JAK-STAT signaling, which plays a critical role in the induction of the cellular response. IL27 is expressed by antigen-presenting cells (APCs) that have been exposed to inflammatory stimuli and causes both pro-inflammatory and anti-inflammatory responses, which influence the immune system in various ways [55–57]. In monocytes, IL27 signals through STAT1, STAT3, and NF-κB [58], whereas, in macrophages, it signals through STAT1 and STAT3 [59]. IL27 has received significant attention due to studies showing that IL27 suppresses replication of viruses, including HIV-1 [60], hepatitis B virus (HBV) [61], hepatitis C virus (HCV) [62], influenza A virus (IAV) [63], cytomegalovirus (CMV) [64], and CHIKV [17, 65]. Recently, we reported that IL27 induces a STAT1-dependent proinflammatory and antiviral response in CHIKV-infected macrophages through two signal pathways: an early signal dependent on recognition of CHIKV-PAMPs by TLR1/2-MyD88 to activate NF-κB, which in turn induces the production of EBI3 mRNA, and a second signal dependent on the recognition of a CHIKV replication intermediate (dsRNA) by TLR3-TRIF to activate IRF1, which induces production of IL27p28 mRNA [17]. Both signaling pathways are required for the production of functional IL27 protein involved in the induction of ISGs, including AVPs, cytokines, and CC and CXC chemokines, in an IFN-independent manner.

Since we and others have reported that IL27 induces a strong response to viral infections in an IFN-independent manner, in the present study we investigated transcription levels of IL27 in COVID-19 patients to determine whether IL27 might induce an antiviral response to SARS-CoV-2 infection. For this, we reanalyzed two mRNA datasets, the first of which was obtained using peripheral blood mononuclear cells (PBMCs) from healthy individuals and patients with moderate, severe, or critical COVID-19 and the second of which was obtained using monocytes from healthy individuals and patients with moderate or severe COVID-19. In addition, we also reanalyzed an mRNA dataset obtained from macrophages stimulated with the SARS-CoV-2 spike protein. Our analysis focused on IL27 signaling components and ISG-encoding genes. This should facilitate a better understanding of the protective and pathogenic immune response in COVID-19 patients.

## Materials and methods

### Bioinformatic analysis of previously published mRNA datasets and prediction of cell signaling pathway activation

To investigate the transcription level of IL27 in COVID-19 patients, we reanalyzed two RNA-Seq datasets. The first RNA-Seq analysis, published by Hadjadj et al. [46] (kindly provided by Terrier B), was performed using PBMCs from healthy individuals (n = 13) and patients with moderate (n = 11), severe (n = 10), or critical (n = 11) COVID-19. The second RNA-Seq dataset (GSE176290) was downloaded from the Genome Expression Omnibus (GEO) [66], and the analysis was performed using monocytes from healthy individuals (n = 9) and patients with moderate (n = 9) or severe (n = 11) COVID-19. We also reanalyzed the publicly available RNA-Seq dataset GSE173488 (GEO) [67], which was obtained using human monocyte-derived macrophages (MDMs) from healthy donors that were either stimulated with 1 µg of SARS-CoV-2 spike protein per ml for 4 hours (n = 4) or not stimulated. Gene expression (mRNA) was expressed as RNA counts, and the count matrix of the RNA-Seq datasets was normalized to counts per million (CPM). Then, mRNA was further normalized by calculating the reads per kilobase per million mapped reads (RPKM) (CPM/gene size [kb]). We detected the activation of cell signaling pathways by quantifying mRNA expression (RNA counts or RPKM) of immune biomarkers. These biomarkers are associated with myeloid markers, migration markers, inducers of the antiviral response, the TLR signaling pathway, the IL27 signaling pathway, IRFs, the NF-κB complex, NF-κB target genes, and ISGs, including antiviral proteins (AVPs), STAT1-dependent cytokines, and STAT1-dependent CC and CXC chemokines. This approach was described previously [17].

## Results

### Expression of myeloid and migratory markers in human PBMCs and monocytes are associated with COVID-19 severity

**Fig. 1:**
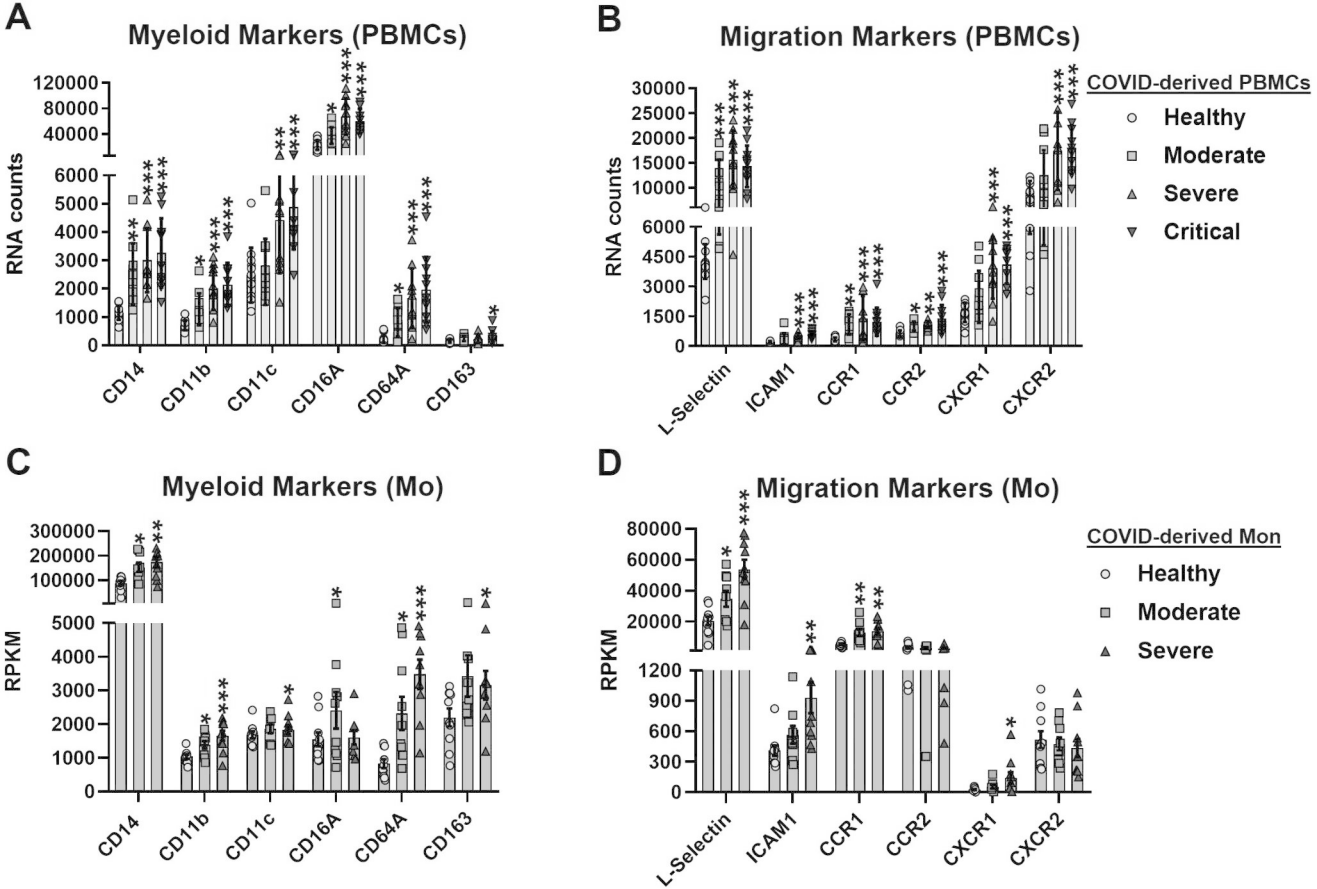
Association of the expression of myeloid and migratory markers in human PBMCs and monocytes with COVID-19 severity. We reanalyzed two mRNA datasets. The first mRNA dataset was published by Hadjadj et al. [46] and was performed using PBMCs from healthy individuals (n = 13) and patients with moderate (n = 11), severe (n = 10), or critical (n = 11) COVID-19. Gene expression (mRNA) was expressed as RNA counts. The second mRNA dataset was the publicly available RNA-Seq GSE176290 (GEO) dataset [66] obtained using monocytes from healthy individuals (n = 9) and patients with moderate (n = 9) or severe (n = 11) COVID-19. Gene expression (mRNA) was normalized by calculating reads per kilobase per million mapped reads (RPKM). (**A**-**D**) Abundance of mRNA encoding myeloid markers (**A** and **C**) and migration markers (**B** and **D**) in COVID-derived PBMCs and COVID-derived monocytes, respectively. Data are presented as the mean ± SD. One-way ANOVA with Fisher’s LSD post-test was performed. Significant differences between healthy individuals and patients with COVID-19 are defined as *p* < 0.05 (*),*p* < 0.01 (**), and *p* < 0.002 (***)

Monocytes are phagocytic cells of the innate immune system that are involved in the immunopathogenesis of viral infection. Zheng et al. reported a significant reduction in monocyte frequency in PBMCs from COVID-19 patients [30], suggesting possible migration of monocytes into the tissues. However, role of human monocytes in immunopathogenesis of SARS-CoV-2 infection is poorly understood. In this study, we evaluated expression of myeloid and migration markers in both COVID-derived PBMCs and COVID-derived monocytes. We observed that both PBMCs and monocytes had upregulated expression of mRNAs of myeloid markers, including CD14, α-integrins (CD11b and CD11c), FCγ receptors (CD64A), and macrophage scavenger receptors (CD163), as a function of the severity of COVID-19 (Fig. 1A and C). Furthermore, COVID-derived PBMCs and monocytes had upregulated expression of migration markers, including endothelial adhesion molecules, including L-selectin and intercellular adhesion molecule 1 (ICAM1), and CC/CXC chemokine receptors (CCR1, CXCR1), as a function of the severity of COVID-19 (Fig. 1B and D). Together, the results show that upregulation of myeloid and migration markers is consistent in both COVID-derived PBMCs and COVID-derived monocytes, suggesting that monocytes could be the main subset of PBMCs that respond to SARS-CoV-2 infection in humans and that their activation might play an immunopathogenic role in SARS-CoV-2 infection.

### mRNAs encoding interleukin 27 subunits are strongly induced in PBMCs and monocytes from COVID-19 patients and are associated with disease severity.

**Fig. 2:**
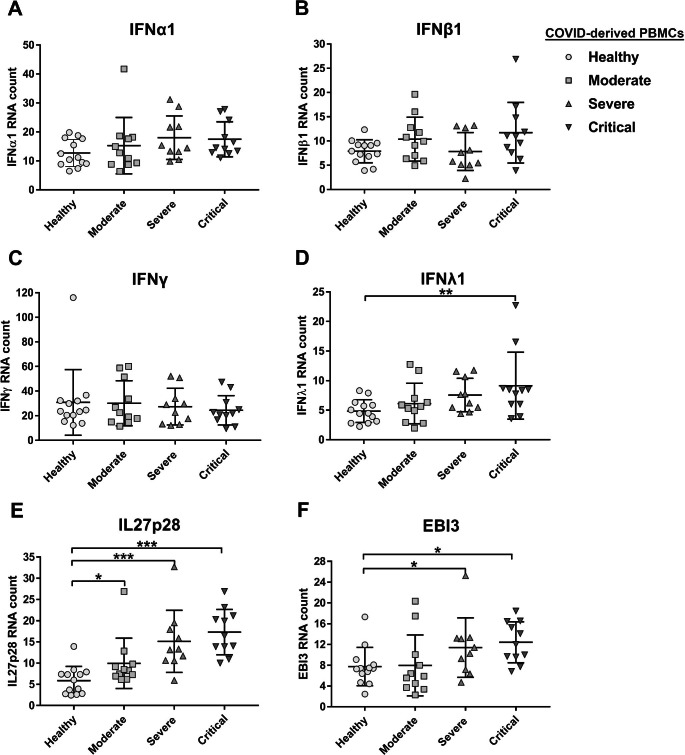
Expression of interleukin 27 and IFNλ1 in PBMCs from COVID-19 patients with different levels of disease severity. We reanalyzed the mRNA dataset published by Hadjadj et al. [46] obtained using PBMCs from healthy individuals (n = 13) and patients with moderate (n = 11), severe (n = 10), or critical (n = 11) COVID-19, using a Nanostring Human Immunology 114 Kit v2. Gene expression (mRNA) was expressed as RNA counts. (**A**-**F**) Abundance of mRNA encoding IFNα1 (**A**), IFNβ1 (**B**), IFNγ (**C**), IFNλ1 (**D**), IL27p28 (**E**), and EBI3 (**F**) in COVID-derived PBMCs. Data are presented as the mean ± SD. One-way ANOVA with Fisher’s LSD post-test was performed. Significant differences between healthy individuals and patients with COVID-19 are defined as *p* < 0.05 (*),*p* < 0.01 (**), and *p* < 0.002 (***)

**Fig. 3:**
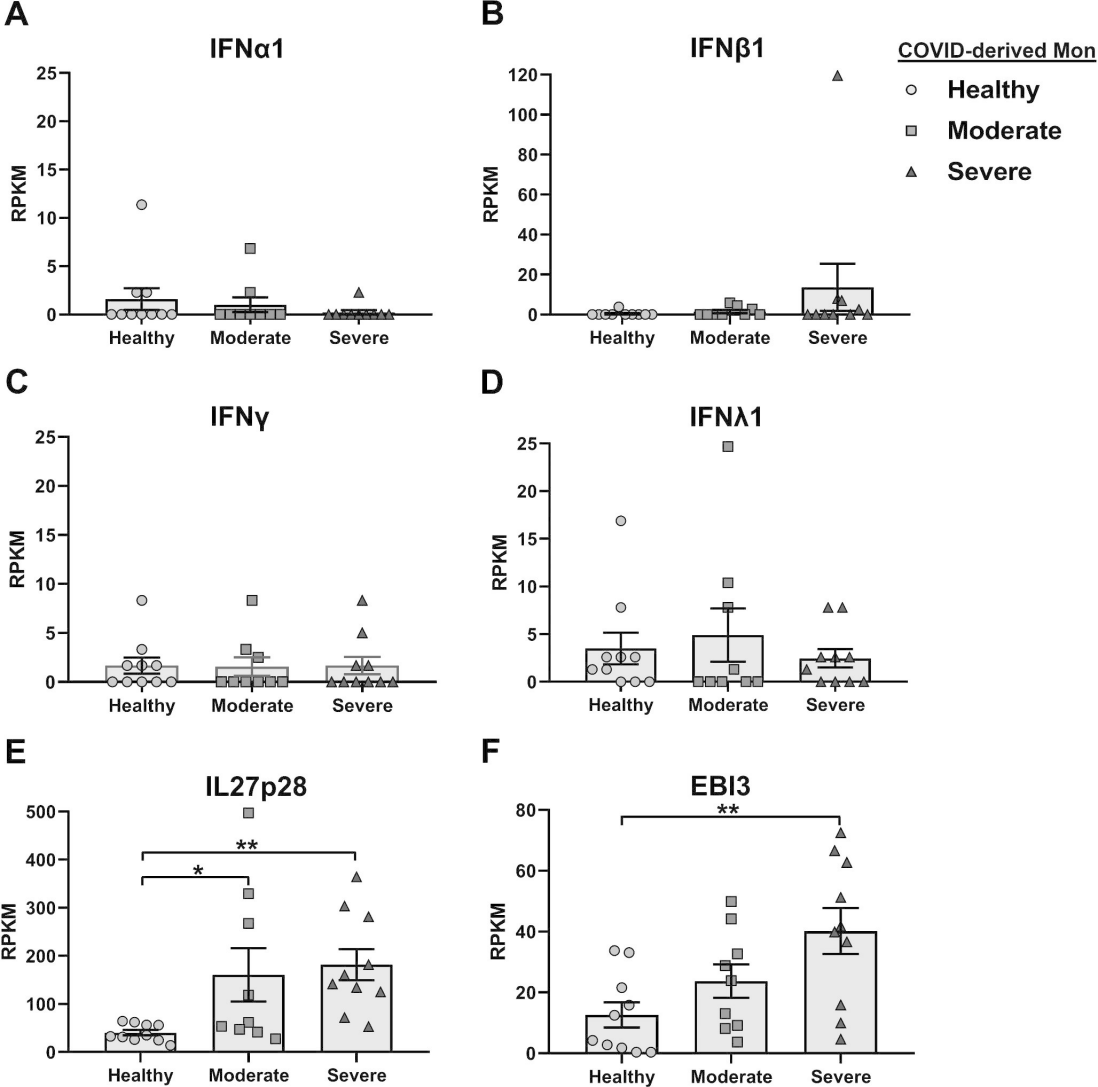
Expression of interleukin 27 in monocytes from COVID-19 patients with different levels of disease severity. We reanalyzed the publicly available RNA-Seq dataset GSE176290 (GEO) [66], which was obtained using monocytes from healthy individuals (n = 9) and patients with moderate (n = 9) or severe (n = 11) COVID-19. Gene expression (mRNA) was normalized by calculating reads per kilobase per million mapped reads (RPKM). (**A**-**F**) Abundance of mRNAs encoding IFNα1 (**A**), IFNβ1 (**B**), IFNγ (**C**), IFNλ1 (**D**), IL27p28 (**E**), and EBI3 (**F**) in COVID-derived monocytes. Data are presented as the mean ± SD. One-way ANOVA with Fisher’s LSD post-test was performed. Significant differences between healthy individuals and patients with COVID-19 are defined as *p* < 0.05 (*), *p* < 0.01 (**), and *p* < 0.002 (***)

Previous reports have shown that SARS-CoV-2 infection antagonizes the IFN-dependent antiviral response [46–48]. In addition, Hadjadj et al. reported that PBMCs from COVID-19 patients did not express significantly high levels of any of the three types of IFN [46]. Here, we measured the expression of mRNA encoding IFNs and IL27 subunits in PBMCs and monocytes from COVID-19 patients. Transcriptomic analysis showed that neither COVID-derived PBMCs nor COVID-derived monocytes expressed IFN-I. This included IFNα1 and IFNβ1 (Figs. 2A and B and 3A and B, respectively), IFN-II, such as IFNγ (Figs. 2C and 3C, respectively), and IFN-III, except for IFNλ1, which was expressed in COVID-derived PBMCs from critical COVID-19 patients (Fig. 2D), but not in COVID-derived monocytes (Fig. 2D). Next, we investigated whether IL27 was positively correlated with COVID-19 severity. Transcriptomic analysis showed that the expression of both IL27 subunits – IL27p28 and EBI3 – increased with severity of COVID-19 in both PBMCs and monocytes (Fig. 2E and F and 3E and F, respectively). Collectively, the data suggest that an increase in IL27 expression correlated with COVID-19 severity, indicating that IL27 might be a marker of the antiviral response to SARS-CoV-2 infection or a possible biomarker of COVID-19 progression.

### Interleukin 27 signaling components are expressed in COVID-derived PBMCs and monocytes

**Fig. 4:**
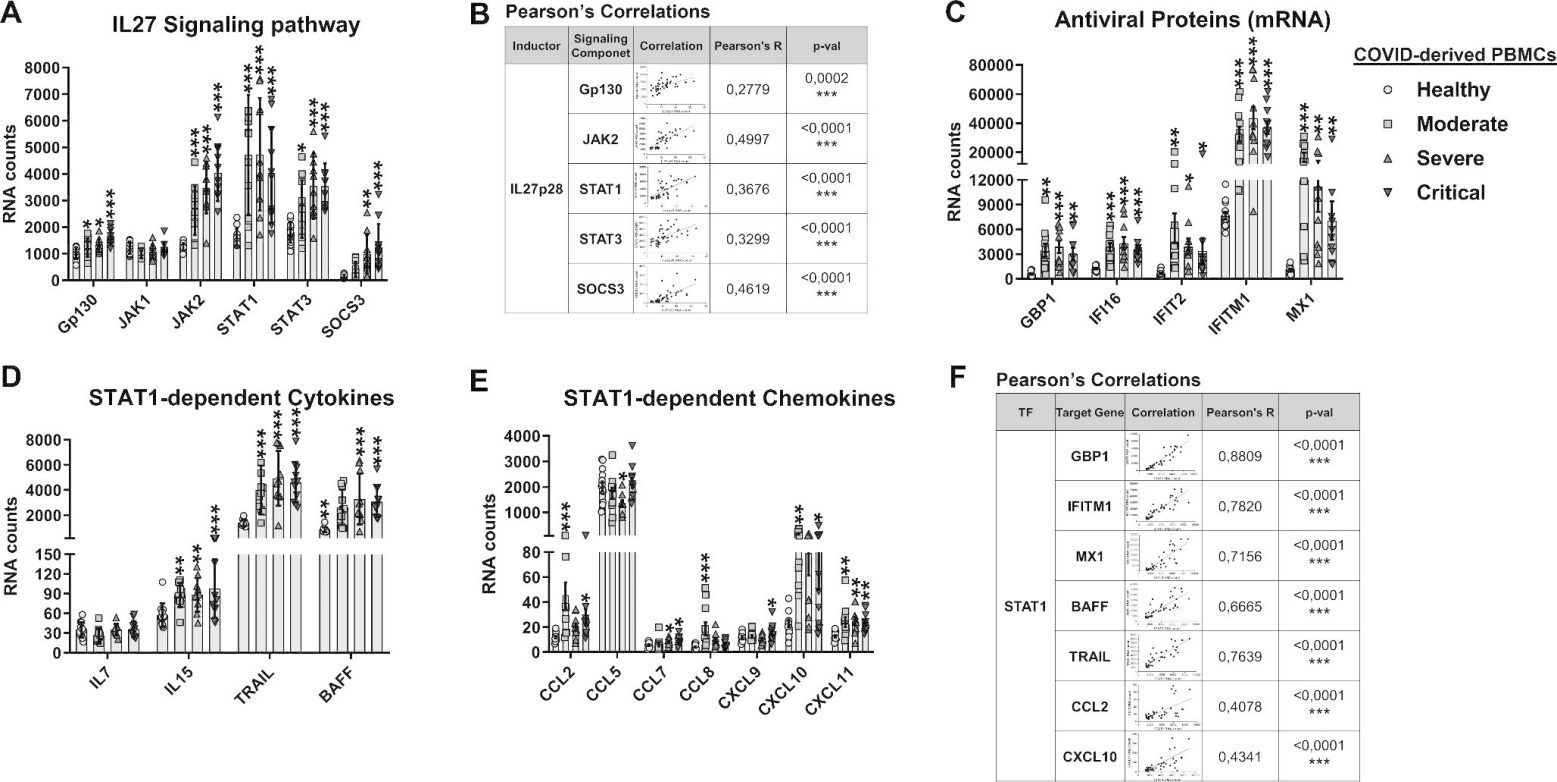
Activation of the interleukin 27 signaling pathway is in COVID-derived PBMCs. We reanalyzed the mRNA dataset published by Hadjadj et al. [46], which was obtained using PBMCs from healthy individuals (n = 13) and patients with moderate (n = 11), severe ( n = 10), or critical (n = 11) COVID-19, using a Nanostring Human Immunology 114 Kit v2. Gene expression (mRNA) was expressed as RNA counts. (**A**) mRNAs abundance of IL27 signaling pathway components in COVID-derived PBMCs. Data are presented as the mean ± SD. One-way ANOVA with Fisher’s LSD post-test was performed. Significant differences between healthy individuals and patients with COVID-19 are defined as *p* < 0.05 (*), *p* < 0.01 (**), and *p* < 0.002 (***). (**B**) Pearson’s correlation between IL27p28 and IL27 signaling pathway components (Gp130, JAK2, STAT1, STAT3, and SOCS3) mRNA abundance in PBMCs from healthy individuals and patients with COVID-19. Significant results are defined as *p* < 0.05 (*), *p* < 0.01 (**), and *p* < 0.002 (***). (**C**-**E**) Abundance of mRNA encoding antiviral proteins (**C**), STAT1-dependent cytokines (**D**), and STAT1-dependent chemokines (**E**) in COVID-derived PBMCs. Data are presented as the mean ± SD. Statistical analysis was performed as described above. (**F**) Pearson’s correlation for comparison of the abundance of mRNA encoding STAT1 and STAT1-target genes (GBP1, IFITM1, MX1, BAFF, TRAIL, CCL2, and CXCL10) mRNA abundance in PBMCs from healthy individuals and patients with COVID-19. Significant results are defined as *p* < 0.05 (*), *p* < 0.01 (**), and *p* < 0.002 (***)

**Fig. 5:**
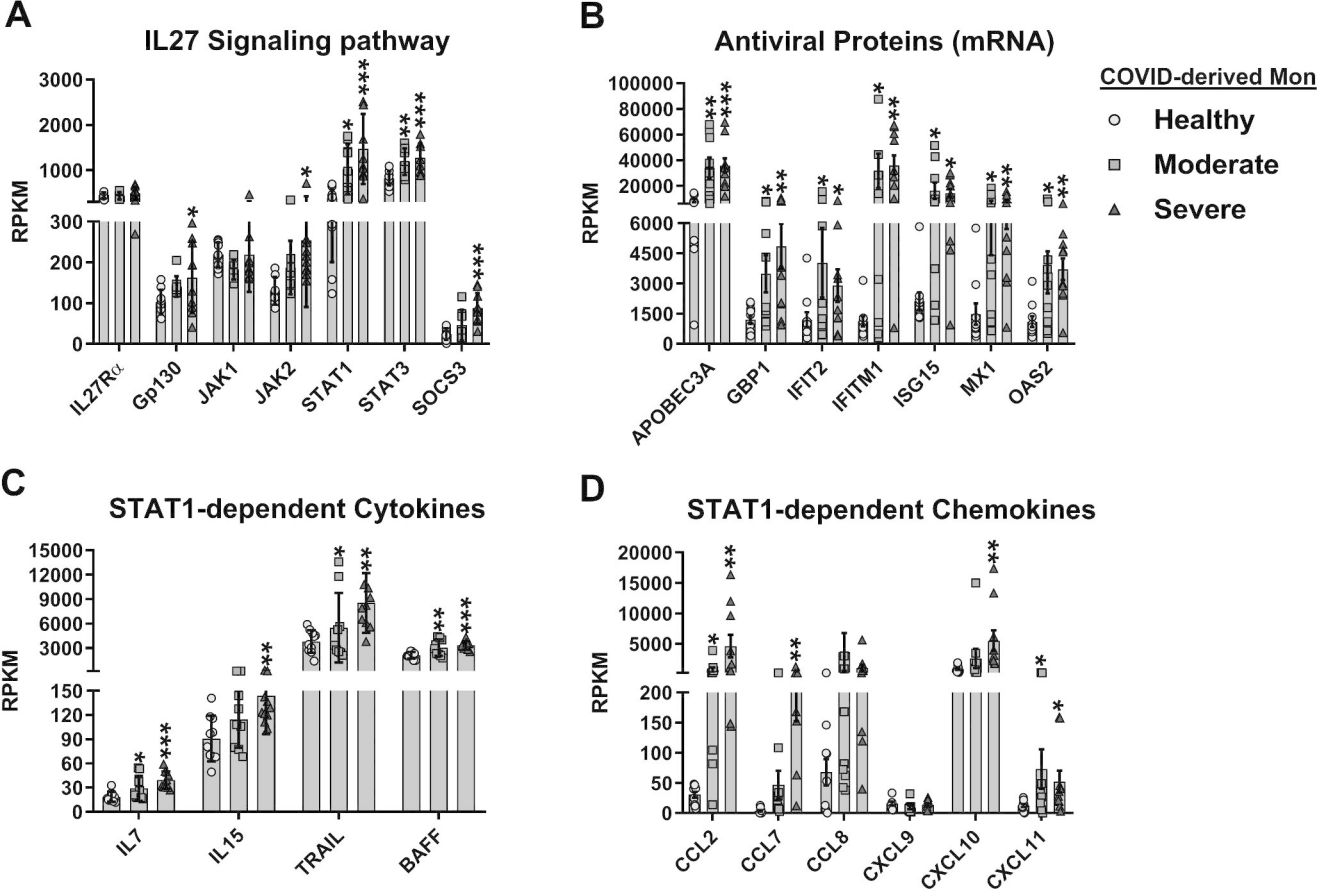
Activation of the interleukin 27 signaling pathway in COVID-derived monocytes. We reanalyzed the publicly available RNA-Seq database GSE176290 (GEO) [66], which was obtained using monocytes from healthy individuals (n = 9) and patients with moderate (n = 9) or severe (n = 11) COVID-19. Gene expression (mRNA) was normalized by calculating reads per kilobase per million mapped reads (RPKM). (**A**-**D**) mRNA abundance of IL27 signaling pathway components (**A**), antiviral proteins (**B**), STAT1-dependent cytokines (**C**), and STAT1-dependent chemokines (**D**) in COVID-derived monocytes. Data are presented as the mean ± SD. One-way ANOVA with Fisher’s LSD post-test was performed. Significant differences between healthy individuals and patients with COVID-19 are defined as *p* < 0.05 (*), *p* < 0.01 (**), and *p* < 0.002 (***)

Considering that COVID-derived PBMCs and monocytes show high levels of both IL27 subunits, we evaluated mRNA expression levels of IL27 signaling components in these cells. We observed that COVID-derived PBMCs and monocytes showed significantly increased levels of receptor subunit Gp130, the receptor-associated kinase JAK2, STAT1, and STAT3, and the negative regulator SOCS3 (Fig. 4A and 5A, respectively) as a function of COVID-19 disease severity. As shown in Fig. 4B, we observed a significant positive correlation between the expression level of mRNA encoding the IL27p28 subunit and IL27 signaling components in COVID-derived PBMCs, including Gp130 (R = 0.2779, *P* = 0.0002), JAK2 (R = 0.4997, *P* < 0.0001), STAT1 (R = 0.3676, *P* < 0.0001), STAT3 (R = 0.3299, *P* < 0.0001), and SOCS3 (R = 0.4619, *P* < 0.0001).

Since we and others have reported that IL27 is essential for antiviral immunity [65, 68], we proceeded to determine ISG mRNA levels in both COVID-derived PBMCs and monocytes. While the expression of guanylate-binding protein 1 (GBP1), interferon-gamma inducible protein 16 (IFI16), interferon-induced protein with tetratricopeptide repeats 2 (IFIT2), interferon-induced transmembrane protein 1 (IFITM1), and MX dynamin-like GTPase 1 (MX1) was increased in COVID-19-derived PBMCs (Fig. 4C), the expression of APOBEC3A, GBP1, IFIT1, IFITM1, ISG15, MX1, and OAS1 was increased in COVID-19-derived monocytes (Fig. 5B). Similarly, we found that the expression pattern of STAT1-dependent cytokines and chemokines, including IL15, TNF-related apoptosis-inducing ligand (TRAIL), and B cell activating factor (BAFF), CCL2, CCL7, CXCL10, and CXCL11 in both PBMCs and monocytes increased with the severity of COVID-19, while CCL8 mRNA was significantly expressed in moderate COVID-19 patients but decreased in severe and critical COVID-19 patients. CXCL9 was significantly expressed in critical COVID-19 patients, but not in other groups (Fig. 4E). As shown in Fig. 4F, a moderately high positive correlation between mRNA expression of STAT1 and its target genes, including ISGs such as GBP1 (R = 0.8809, *P* < 0.0001), IFITM1 (R = 0.7820, *P* < 0.0001), MX1 (R = 0.7156, *P* < 0.0001); cytokines such as BAFF (R = 0.6665, *P* < 0.0001) and TRAIL (R = 0.7639, *P* < 0.0001), and chemokines, including CCL2 (R = 0.4078, *P* < 0.0001), and CXCL10 (R = 0.4341, *P* < 0.0001) was observed in COVID-derived PBMCs. The data suggest that IL27 signaling was activated in COVID-derived PBMCs and monocytes to induce a robust STAT1-dependent pro-inflammatory and antiviral response that was dependent on ISG expression.

### Expression levels of TLRs, TLR adapter, and TLR downstream signaling components are associated with the severity of COVID-19.

**Fig. 6:**
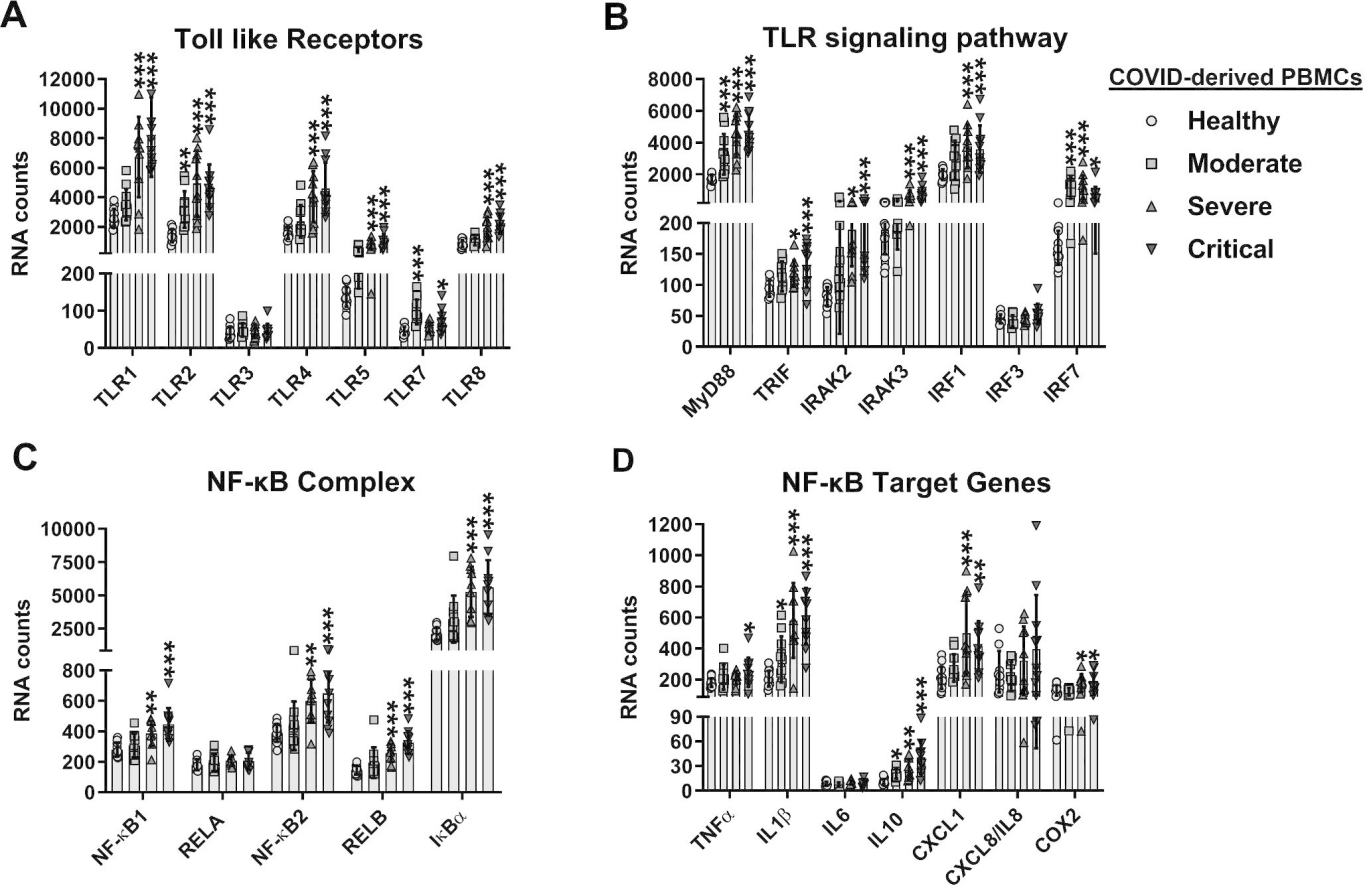
Association of activation of the Toll-like receptor signaling pathway with induction of interleukin 27 expression in PBMCs from COVID-19 patients. We reanalyzed the mRNA dataset published by Hadjad et al. [46], which was obtained using PBMCs from healthy individuals (n = 13) and patients with moderate (n = 11), severe (n = 10), or critical (n = 11) COVID-19, using a Nanostring Human Immunology 114 Kit v2. Gene expression (mRNA) was expressed as RNA counts. (**A**-**D**) mRNAs abundance of Toll-like receptors (**A**), TLR signaling pathway (**B**), NF-κB complex (**C**), and NF-κB target genes (**D**) in COVID-derived PBMCs. Data are presented as the mean ± SD. One-way ANOVA with Fisher’s LSD post-test was performed. Significant differences between healthy individuals and patients with COVID-19 are defined as *p* < 0.05 (*), *p* < 0.01 (**), and *p* < 0.002 (***)

**Fig. 7:**
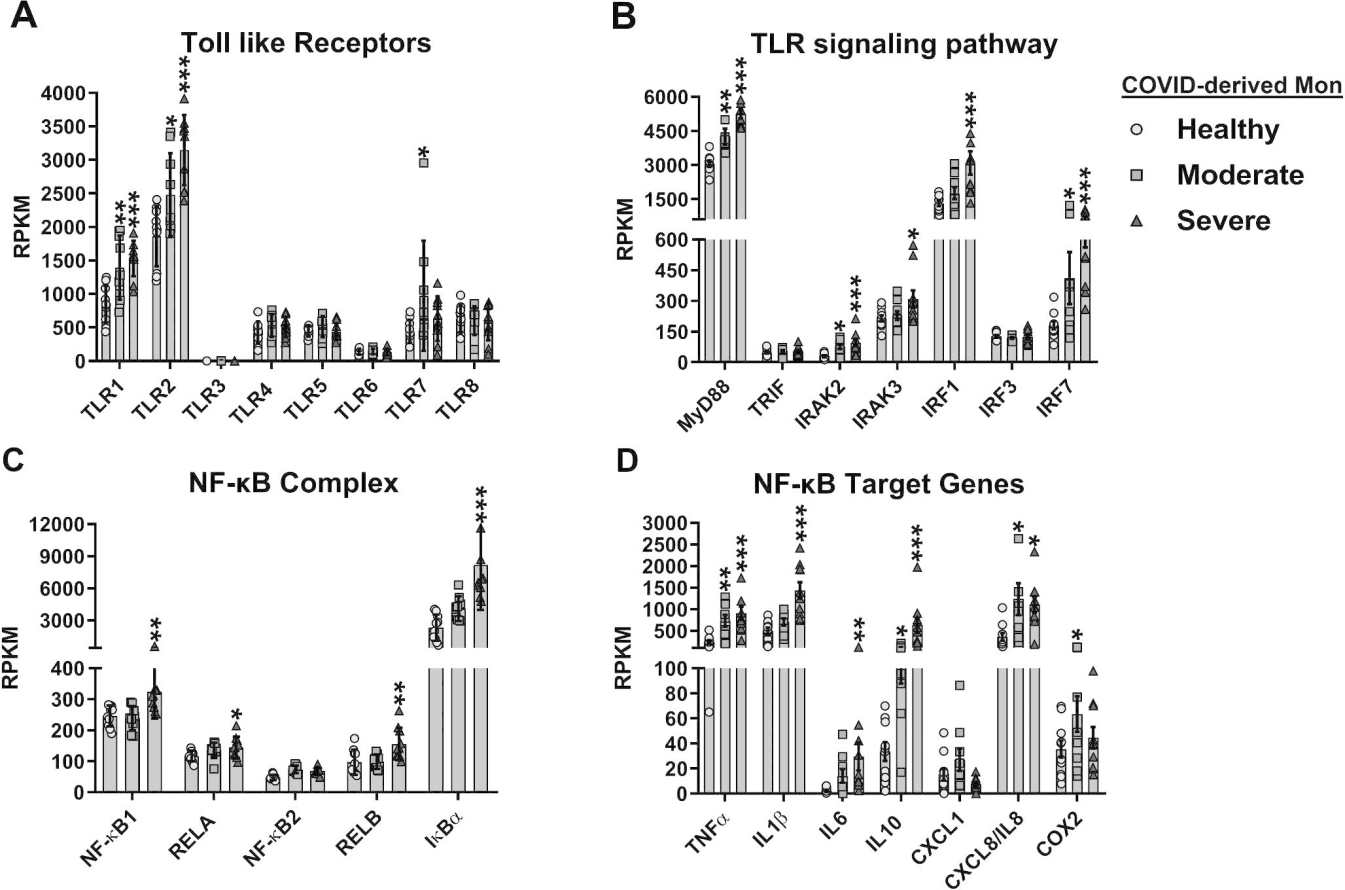
Association of activation of the Toll-like receptor signaling pathway with induction of interleukin 27 expression in monocytes from COVID-19 patients. We reanalyzed the publicly available RNA-Seq dataset GSE176290 (GEO) [66], obtained using monocytes from healthy individuals (n = 9) and patients with moderate (n = 9) or severe (n = 11) COVID-19. Gene expression (mRNA) was normalized by calculating reads per kilobase per million mapped reads (RPKM). (**A**-**D**) mRNA abundance of Toll-like receptors (**A**), TLR signaling pathway (**B**), NF-κB complex (**C**), and NF-κB target genes (**D**) in COVID-derived monocytes. Data are presented as the mean ± SD. One-way ANOVA with Fisher’s LSD post-test was performed. Significant differences between healthy individuals and patients with COVID-19 are defined as *p* < 0.05 (*), *p* < 0.01 (**), and *p* < 0.002 (***)

TLR activation drives transcriptional expression of the IL27p28 and EBI3 genes through the induction of IRF1 and NF-κB, respectively [17]. To determine whether the expression of TLRs was positively correlated with COVID-19 severity, we analyzed the dataset for TLR expression in patients with differing severities of COVID-19 (Figs. 6 and 7). We found that the expression of TLR2 increased with the severity of COVID-19 in both PBMCs and monocytes (Figs. 6A and 7A, respectively), similar to what was observed with IL27 subunits (Fig. 2E and F). We noted that TLR1 in monocytes also increased with the severity of COVID-19 (Fig. 7A). In addition, the expression of TLR1, TLR4, TLR5, and TLR8 was significantly elevated in patients with severe or critical COVID-19, and the expression of TLR7 was increased in patients with moderate or critical COVID-19 (Fig. 6A). By contrast, expression of TLR4, TLR5, TLR6, and TLR7 was not altered in COVID-19-derived monocytes (Fig. 7A). The expression of TLR3 did not show changes with the progression of COVID-19 in either PBMCs or monocytes (Figs. 6A and 7A). The data suggest a link between IL27 and certain TLRs and disease progression in patients with COVID-19.

MyD88 is important for proinflammatory cytokine production during SARS-CoV-1 infection [69]. To determine whether MyD88 or another TLR adapter, TRIF (TIR-domain-containing adapter-inducing interferon-β), plays a role in SARS-CoV-2-induced inflammatory responses, we analyzed mRNA expression of these factors in PBMCs and monocytes from patients with differing severity of COVID-19. We found that the expression of MyD88 in both PBMCs and monocytes increased with the severity of COVID-19 (Figs. 6B and 7B). By contrast, the expression of TRIF was significantly increased only in PBMCs from patients with severe or critical COVID-19, but not in monocytes (Figs. 6B and 7B). Furthermore, as shown in Fig. 6B, mRNA levels of key mediators of TLR signaling, including IRAK2 and IRAK3, were increased in PBMCs from patients with severe or critical COVID-19. Also, while IRAK2 was elevated in monocytes from patients with moderate or severe COVID-19, IRAK3 was only increased in severe COVID-19 (Fig. 7B).

Although our transcriptomic analysis showed no expression of IFNs, we proceeded to evaluate the expression of IRFs, since they are induced by signaling via PRRs, including TLRs. We found that the expression pattern of IRF7 in both PBMCs and monocytes increased with the severity of COVID-19 (Figs. 6A and 7A). While the expression of IRF1 was significantly increased in PBMCs from patients with severe or critical COVID-19 (Fig. 6B), its expression in monocytes was increased only in patients with severe COVID-19 (Fig. 7B), as was observed with IL27p28 and EBI3 (Fig. 2E and F and Fig. 3E and F). IRF3 expression in PBMCs and monocytes of COVID-19 patients did not show any changes with disease progression (Fig. 6A and 7B, respectively). Levels of mRNA encoding various components of the NF-κB complex, including NF-κB1, NF-κB2, RELB, and IκBα, were significantly elevated in PBMCs from patients with severe or critical COVID-19 (Fig. 6C). In contrast, these components were increased only in monocytes from patients with severe COVID-19 (Fig. 7C). Similarly, mRNA levels of NF-κB target genes, including IL1β and IL10, were significantly increased independently of the severity of COVID-19, and CXCL1 and cyclooxygenase 2 (COX2) mRNA expression was significantly higher in PBMCs from patients with severe and critical COVID-19 (Fig. 6D). TNFα mRNA expression was significantly increased only in critical-COVID-19-derived PBMCs (Fig. 6D). In COVID-19-derived monocytes, TNFα, IL1β, IL6, IL10, CXCL8/IL8, and COX2 were also significantly increased, depending on disease severity (Fig. 7D). Collectively, the results shown here suggest an association of TLRs with the pathogenesis of COVID-19.

### The SARS-CoV-2 spike protein modulates expression of TLR1/2-MyD88 and downstream signaling components and induces IL27-dependent pro-inflammatory and antiviral responses in human macrophages

**Fig. 8:**
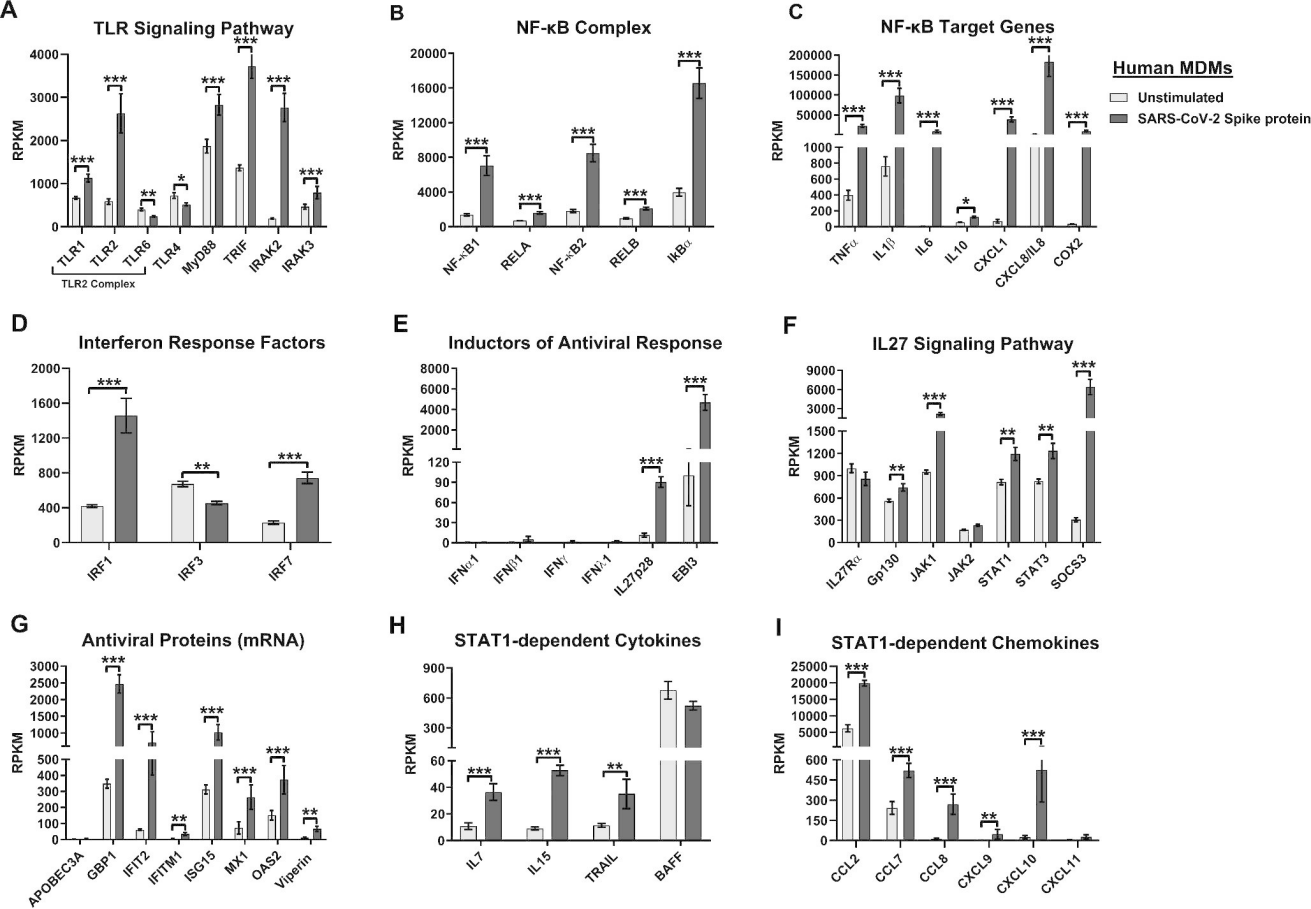
Modulation of TLR1/2 signaling components by the SARS-CoV-2 spike protein and induction of a robust IL27-dependent pro-inflammatory and antiviral response in human macrophages. We reanalyzed the publicly available RNA-Seq dataset GSE173488 (GEO) [67], which was obtained using human MDMs from healthy donors that were stimulated with 1 µg of SARS-CoV-2 spike protein per ml for 4 hours or not stimulated. Gene expression (mRNA) was normalized by calculating reads per kilobase per million mapped reads (RPKM). (**A**-**I**) mRNA abundance of the TLR signaling pathway (**A**), NF-κB complex (**B**), NF-κB target genes (**C**), interferon response factors (**D**), inducers of the antiviral response (**E**), the IL27 signaling pathway (**F**), antiviral proteins (**G**), STAT1-dependent cytokines (**H**), and STAT1-dependent chemokines (**I**) MDMs in SARS-CoV-2 stimulated with the spike protein. Data are presented as the mean ± SD. A *t*-test was performed. Significant differences are defined as *p* < 0.05 (*), *p* < 0.01 (**), and *p* < 0.002 (***). n = 4

Both TLR2 and MyD88 expression are associated with the severity of COVID-19 disease, since they induce the production of pro-inflammatory cytokines by sensing structural PAMPs of SARS-CoV-2 [30]. Furthermore, activation of TLR1/2-MyD88 signaling induces a robust NF-κB-dependent pro-inflammatory response and a low but significant IL27-dependent antiviral response in both human and murine macrophages [17]. To determine whether TLR1/2-MyD88 induction by SARS-CoV-2 spike protein (S protein) plays a role in the establishment of the IL27-dependent pro-inflammatory and antiviral response in macrophages, we reanalyzed a publicly available RNA-seq dataset, GSE173488 (GEO) [67]. As shown in Fig. 8A, transcriptomic analysis showed that human MDMs stimulated with the SARS-CoV-2 S protein significantly upregulated TLR1 and TLR2 mRNA, as well as TLR adapters and mediators, including MyD88, TRIF, IRAK2, and IRAK3, as found in COVID-derived PBMCs and monocytes. These results are consistent with the significantly high expression levels of NF-κB complex components, including NF-κB1, NF-κB2, RELA, RELB, and IκBα (Fig. 8B), as well as genes targeted by NF-κB (TNFα, IL1β, IL6, IL10, CXCL1, CXCL8/IL8, and COX2; Fig. 8C). We also found that IRF1 and IRF7 mRNA levels increased significantly in MDMs stimulated with SARS-CoV-2 S protein, whereas expression of IRF3 was downregulated (Fig. 8D). Interestingly, while MDMs stimulated with SARS-CoV-2 S protein do not produce IFN-I (IFNα1 and IFNβ1), IFN-II (IFNγ), and IFN-III (IFNλ1) mRNAs, significantly increased levels of IL27p28 and EBI3 mRNA were observed (Fig. 8E). Also, we found that the levels of IL27 signaling components (Gp130, JAK1, STAT1, STAT3, and SOCS3) were significantly elevated in MDMs stimulated with SARS-CoV-2 S protein (Fig. 8F). As expected, we found significantly increased expression of AVP-encoding ISG mRNAs, including GBP1, IFIT2, IFITM1, ISG15, MX1, OAS2, and viperin, as well as STAT1-dependent cytokines such as IL7, IL15, and TRAIL, in MDMs stimulated with the SARS-CoV-2 S protein (Fig. 8G-I). Overall, the data suggest that stimulation of human MDMs with SARS-CoV-2 S protein induces TLR1/2-MyD88 signaling, which in turn promotes transcription of the genes encoding both IL27 subunits – IL27p28 and EBI3 – leading to a strong IL27-dependent pro-inflammatory and antiviral response in an IFN-independent manner.

## Discussion

Recognition of viral components by innate immune receptors such as PRRs activates both IFN-I and IFN-III responses [70]. Both TLRs and RIG-I/MDA5 are activated in response to viral infection and initiate signaling cascades to induce IFNα/β and IFNλ [70, 71], which induces ISGs, the main effectors of antiviral protection [72]. There is an emerging body of evidence showing that SARS-CoV-2 infection elicits weaker induction of IFN-I and a robust inflammatory response in COVID-19 patients [46]. This loss of IFN-I-related immunity might result in severe symptoms in patients with COVID-19 [53]. Other studies have suggested that elevated IFNs levels correlate with and contribute to severe COVID-19 [73–75]. It has been proposed that severe COVID-19 is associated with uncontrolled IFN-I production, leading to pathology instead of viral containment [76]. Although SARS-CoV-2 has evolved multiple mechanisms to block the induction and action of IFNs [77, 78], and despite the fact that IFN-I levels in patients’ sera are below detectable levels, ISG expression can be detected [46, 79]. Thus, the results suggest that either a limited amount of IFN is sufficient to induce ISGs or, alternatively, the expression of ISGs is activated in an IFN-independent manner in COVID-19 patients. In the current study, we performed a transcriptomic analysis of PBMCs and monocytes from COVID-19 patients classified as moderate, severe, or critical. We did not observe changes in IFN-I (IFNα and IFNβ), IFN-II (IFNγ), or IFN-III (IFNλ1) mRNA levels in COVID-derived PBMCs or monocytes, except for IFNλ1, which was significantly increased in PBMCs from patients with critical COVID-19. Importantly, we noted that the levels of mRNA encoding IL27p28 and EBI3, i.e., both IL27 subunits, increased significantly with the severity of COVID-19. Thus, PBMCs from COVID-19 patients in critical condition or monocytes from COVID-19 patients in severe condition showed the highest expression levels of the two IL27 subunits. In agreement with our results, Zamani et al. reported that the levels of IL27 were significantly higher in COVID-19 patients than in healthy subjects and that disease severity was associated with the IL27 level [80]. While IL27 was significantly reduced in severe COVID-19 patients who required intensive care, the IL27 level was significantly higher in severe COVID-19 survivors [80]. In addition, it was reported previously that IL27 levels in serum were higher in patients with chikungunya fever than in those in the acute or subacute stage of the disease [81]. In influenza A virus infection, delivery of recombinant IL27 was shown to limit the recruitment of inflammatory cells, including monocytes and neutrophils, into the lung, without affecting the T cell response, suggesting that IL27 can control the immune response to influenza virus infection [82]. By contrast, low levels of IL27 have been reported in COVID-19 patients at admission [83]. When the authors compared mild, moderate, and severe cases, they did not observe statistically significant differences in the levels of cytokines, including IL27. The authors proposed IL27 as an early biomarker of COVID-19 severity, as was proposed previously by Cavalcanti et al. for patients with chikungunya fever [81]. In agreement with our transcriptomic analysis, other studies described high levels of IL27 in COVID-19 patients. Huang et al. reported significant elevation of IL27 in children with multisystem inflammatory syndrome, an acute, febrile and severe SARS-CoV-2-associated syndrome, as compared to controls [84]. Another study of COVID-19 patients also found an increase in IL27 production [85]. *In vitro* studies using human aortic endothelial cells treated with recombinant SARS-CoV-2 S protein resulted in enhanced secretion of inflammatory cytokines, including IL27 [86]. Based on these observations, together with our results showing a correlation with the severity of the disease, we propose that IL27 can be a useful marker of COVID-19 disease progression.

Both PBMCs and monocytes from COVID-19 patients classified according to their severity express high levels of the two subunits of IL27, which is dependent on the severity of the disease. The level of mRNA encoding STAT1 and STAT3 is elevated in COVID-19 patients, regardless of disease severity, and the results suggest that IL27 signaling is activated in response to SARS-CoV-2 infection. This observation could be of importance in altering the inflammatory response, as it has been reported that IL27 acts in both a pro- and anti-inflammatory manner, downregulating the Th17 response via STAT1, while signaling naïve T cells in a STAT3-dependent manner [87]. Likewise, in COVID-derived PBMCs and COVID-derived monocytes, the mRNA expression levels of STAT1-dependent cytokines, as well as CC and CXC chemokines were significantly increased as a function of the severity of disease, consistent with the development of inflammation observed in COVID-19 patients.

A comprehensive understanding of the inflammatory and antiviral response to SARS-CoV-2 infection is needed to allow us to identify their associations with COVID-19 pathogenesis versus protection from SARS-CoV-2 infection. Such information is also critical for identifying alternative pathways that could suppress SARS-CoV-2 infection, given the dysfunction of the IFN signaling pathway described in COVID-19 patients. SARS-CoV-2 evades the IFN-mediated antiviral response with the help of several viral proteins [reviewed in ref. 88]. In addition, the viral nsp16 protein, in conjunction with nsp10, methylates viral mRNAs at the 5′ end to mimic host mRNA and inhibit the activation and function of RIG-I/MDA5, which is involved in the recognition of SARS-CoV-2 RNA [89, 90]. Furthermore, the viral proteins nsp1, nsp3, nsp6, nsp13, orf3a, orf6, orf7a, orf7b, orf8, and M inhibit ISG expression by blocking the activation and nuclear translocation of the ISGF3 complex [reviewed in ref. 88]. Although we did not observe modulation of expression of the genes encoding type I and II IFNs, we did observe significantly higher levels of ISG expression, depending on the severity of the disease, in both COVID-derived PBMCs and monocytes, and also in MDMs treated with the SARS-CoV-2 S protein. In agreement with our results, IL27-dependent ISG expression has been observed in other studies [65, 68]. Generation of innate AVPs, including OAS1, OAS2, OASL, and MX1, in response to IL27 was found to be protective against Zika virus infection in human keratinocytes [68]. This antiviral response occurs in a STAT1- and IRF3-dependent but STAT2-, TYK2-, and IFNAR1-independent manner. Recently, we reported that IL27 signaling was activated in CHIKV-infected MDMs and that the kinetics of IL27p28/EBI3 mRNA expression and IL27 protein production correlated with the expression of AVPs in CHIKV-infected MDMs [65]. Furthermore, we showed that stimulation of THP-1-derived macrophages with recombinant human IL27 induced JAK-STAT signaling and a robust pro-inflammatory and antiviral response in an IFN-independent manner [65]. The current study has shown that expression of ISGs, including APOBEC3A, GBP1, IFIT2, IFITM1, ISG15, MX1, OAS2, and viperin, is driven by IL27 in a STAT1-dependent manner, which was induced through the activation of TLR1/2-MyD88 signaling, although it has previously been shown to be induced primarily by type I IFNs [91]. In agreement with our results, Patel et al. reported that IL27 is expressed by fibroblasts and induces IDO1, APOBEC3G, and MxA expression [92]. Consistent with these results, Pratumchai et al. reported that B-cell-derived IL27 promotes control of persistent lymphocytic choriomeningitis virus (LCMV) infection, and B-cell-derived IL27 drives antiviral immunity and antibody responses by regulating virus-specific CD4^+^ T cells and Tfh cell functions during the late stages of persistent LCMV infection [98]. Consequently, we propose that IL27 also constitutes an alternative line of defense, not only against SARS-CoV-2 but also against other viruses. Our literature review on COVID-19/SARS-CoV-2 revealed a gap in knowledge regarding the potential involvement of IL27 in the induction of ISG expression. We hypothesized that IL27 may play a role in controlling SARS-CoV-2 infection through ISG expression. To the best of our knowledge, this hypothesis has not been addressed in the literature.

We observed increased levels of mRNA encoding TLR1/TLR2 (MyD88-dependent TLRs), as well as the TLR1/2-MyD88 signaling components, NF-κB complex, and IRFs in COVID-derived PBMCs, COVID-derived monocytes, and S-protein-stimulated MDMs. Based on this observation, we hypothesize that recognition of SARS-CoV-2 PAMPs by TLR1/2-MyD88 induces IL27 expression. This, in turn, activates a robust IL27-STAT1-dependent pro-inflammatory and antiviral response in COVID-19 patients that correlates with disease severity. This hypothesis is supported by reports showing that TLR2 recognizes the SARS-CoV-2 envelope and spike proteins to induce an NF-κB-dependent pro-inflammatory response [29–31]. We also observed that, following stimulation of TLR1/2-MyD88 with their respective agonists, human MDMs and murine BMDMs produce IL27 to induce both pro-inflammatory and antiviral responses [17]. Wirtz et al. reported that stimulation of murine DCs via TLR2, TLR4, and TLR9 (MyD88-dependent TLRs) transactivates the EBI3 promoter via MyD88 and NF-κB [93]. Similarly, it has been reported that IL27p28 is highly expressed in murine DCs and peritoneal macrophages in response to TLR3 and TLR4 activation [94, 95]. Zhang et al. reported that IRF1 and IRF8 regulate IL27p28 gene transcription in murine macrophages in response to TLR4 activation by specifically binding to the IRF1 response element in the IL27p28 promoter [96]. Collectively, these studies suggest that regulation of IL27 expression in monocytes, macrophages, and DCs is dependent on TLR signaling and activation of NF-κB and IRF1 to induce transcriptional expression of EBI3 and IL27p28 genes. In the current study, we report that the levels of TLR1/2-MyD88 signaling components, the NF-κB complex, and IRF1 were significantly increased as a function of the severity of COVID-19, and the results were consistent with expression levels of IL27 subunits in COVID-19 patients. Thus, IL27 could play a novel host-defensive role to subvert viral inhibition of IFNs and possibly to take over infection control.

In the current study, we reanalyzed RNA-Seq data obtained using MDMs stimulated with the SARS-CoV-2 S protein [67]. The authors of the original study showed that the SARS-CoV‐2 S protein primes inflammasome formation and the release of mature IL1β in macrophages derived from COVID‐19 patients, but not in macrophages from healthy SARS‐CoV‐2-naïve individuals. Interestingly, our transcriptomic analysis of these MDMs from healthy individuals, stimulated with the SARS-CoV-2 S protein also yielded similar results to those we observed in PBMCs and monocytes from COVID-19 patients. We found that stimulation of MDMs with the SARS-CoV-2 S protein leads to the expression of TLR1/2-MyD88 signaling components, the NF-κB-complex, NF-κB-target genes, IRFs, and both subunits of IL27 (IL27p28 and EBI3), as well as the induction of ISGs, including STAT1-dependent AVPs, cytokines, and chemokines, but not the expression of any type of interferon. Barreda et al. reported that interaction of DCs with the SARS-CoV-2 S protein promotes activation of key signaling molecules involved in inflammation, including MAPK, AKT, STAT1, and NF-κB, which correlates with the production of pro-inflammatory cytokines [97]. Furthermore, the immunopathology induced by the SARS-CoV-2 S protein in macrophages has been shown to be triggered by STAT1 phosphorylation [98]. Collectively, our findings with PBMCs and monocytes derived from COVID‐19 patients and S-protein-stimulated MDMs suggests that SARS-CoV-2 infection causes profound changes in the transcription program that drives an unrecognized mechanism that induces an inflammatory response and acts in a host antiviral response that is IL27-dependent and IFN-independent. Although our study adds to the list of cytokines produced by APCs that are essential for defense against viruses, further study is needed to determine the mechanisms by which IL27 signaling protects the host from SARS-CoV-2 infection.

## Conclusions

**Fig. 9:**
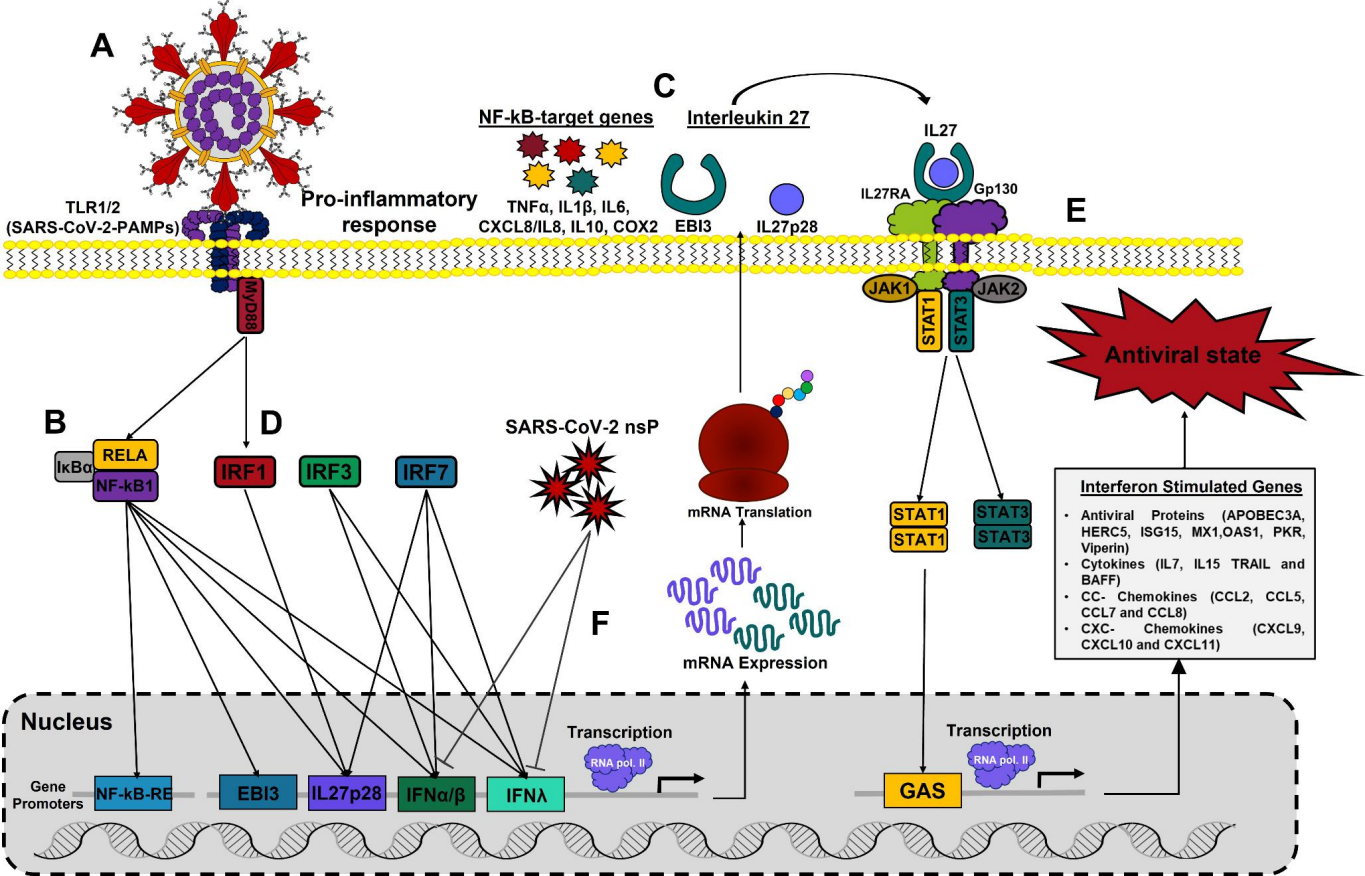
Model. We suggest that recognition of SARS-CoV-2 PAMPs by TLR1/2-MyD88 (**A**) induces NF-κB-complex activation (**B**) and a robust pro-inflammatory response dependent on expression of the NF-κB-target genes (**C**), including EBI3. Induction of TLR1/2-MyD88 signaling by SARS-CoV-2 infection activates IRF1 to induce IL27p28 mRNA expression (**D**) and produce functional IL27, which induces a robust IL27-STAT1-dependent pro-inflammatory and antiviral response associated with induction of the antiviral state in COVID-19 patients (**E**), which correlates with disease severity. Thus, IL27 may be able to trigger a host antiviral response in an IFN-independent manner (**F**)

Transcriptomic analysis of COVID-derived PBMCs, COVID-derived monocytes, and SARS-CoV-2 S-protein-stimulated MDMs revealed significant increases in the levels of mRNA encoding TLR1 and TLR2 (MyD88-dependent TLRs), as well as the TLR1/2-MyD88 signaling components, the NF-κB complex, NF-κB target genes, IRFs, the two IL27 subunits (IL27p28 and EBI3), IL27 signaling components, and ISGs, including STAT1-dependent AVPs, cytokines, and CC and CXC chemokines, but not IFN genes. We suggest that recognition of structural SARS-CoV-2 PAMPs by TLR1/2-MyD88 (Fig. 9A) induces activation of the NF-κB complex (Fig. 9B) and a robust pro-inflammatory response that is dependent on expression of NF-κB target genes (Fig. 9C), including EBI3. Furthermore, induction of TLR1/2-MyD88 signaling by SARS-CoV-2 infection activates IRF1 to induce IL27p28 mRNA expression (Fig. 9D) and produce functional IL27, which induces a robust IL27-STAT1-dependent pro-inflammatory and antiviral response associated with induction of the antiviral state in COVID-19 patients (Fig. 9E), which is associated with disease severity. Thus, IL27 may be able to trigger an antiviral response in an IFN-independent manner (Fig. 9F). Identification of innate antiviral immune factors that are independent of IFN signaling may lead to the development of novel therapeutics against SARS-CoV-2 infection in humans.

## Limitations of the study

A major limitation of this work is that the results obtained by transcriptomic analysis of two mRNA datasets obtained using PBMCs and monocytes from COVID-19 patients classified according to severity grade were not confirmed experimentally, although the validity of these findings was verified indirectly using data obtained with macrophages stimulated with the SARS-CoV-2 S protein. Nevertheless, our analysis allowed us to identify an alternate mechanism that might counteract the ability of SARS-CoV-2 to interfere with the IFN signaling pathway and induction of ISG expression. This should be tested experimentally in an *in vivo* animal model.
